# Initial Medical Attention on Patients with Early-Stage Non-Small Cell Lung Cancer

**DOI:** 10.1371/journal.pone.0032644

**Published:** 2012-03-07

**Authors:** Xing Chen, Ivan P. Gorlov, Jun Ying, Kelly W. Merriman, Marek Kimmel, Charles Lu, Cielito C. Reyes-Gibby, Olga Y. Gorlova

**Affiliations:** 1 Department of Epidemiology, The University of Texas M. D. Anderson Cancer Center, Houston, Texas, United States of America; 2 Department of Genitourinary Medical Oncology, The University of Texas M. D. Anderson Cancer Center, Houston, Texas, United States of America; 3 Department of Statistics, Rice University, Houston, Texas, United States of America; 4 Department of Thoracic/Head and Neck Medical Oncology, The University of Texas M. D. Anderson Cancer Center, Houston, Texas, United States of America; National Taiwan University Hospital, Taiwan

## Abstract

**Background:**

Detection of early stage non-small cell lung cancer (NSCLC) is commonly believed to be incidental. Understanding the reasons that caused initial detection of these patients is important for early diagnosis. However, these reasons are not well studied.

**Methods:**

We retrospectively reviewed medical records of patients diagnosed with stage I or II NSCLC between 2000 and 2009 at UT MD Anderson Cancer Center. Information on suggestive LC-symptoms or other reasons that caused detection were extracted from patients' medical records. We applied univariate and multivariate analyses to evaluate the association of suggestive LC-symptoms with tumor size and patient survival.

**Results:**

Of the 1396 early stage LC patients, 733 (52.5%) presented with suggestive LC-symptoms as chief complaint. 347 (24.9%) and 287 (20.6%) were diagnosed because of regular check-ups and evaluations for other diseases, respectively. The proportion of suggestive LC-symptom-caused detection had a linear relationship with the tumor size (correlation 0.96; with p<.0001). After age, gender, race, smoking status, therapy, and stage adjustment, the symptom-caused detection showed no significant difference in overall and LC-specific survival when compared with the other (non-symptom-caused) detection.

**Conclusion:**

Symptoms suggestive of LC are the number one reason that led to detection in early NSCLC. They were also associated with tumor size at diagnosis, suggesting early stage LC patients are developing symptoms. Presence of symptoms in early stages did not compromise survival. A symptom-based alerting system or guidelines may be worth of further study to benefit NSCLC high risk individuals.

## Introduction

In 2009 and 2010, lung cancer (LC) continued to have the highest incidence and mortality of all cancers [1], [2]. Most cases of LC are found at an advanced stage and thus are associated with a high mortality rate [3]. Recently, National Lung Screening Trial (NLST) has shown that low-dose CT screening results in 20% mortality reduction in individuals at high LC risk. This setting overall reflects the large gap in the early detection of non-small cell LC (NSCLC) that can be addressed.

Unlike advanced LC where detection is thought to be always triggered by symptoms, detection of early stage NSCLC patients is commonly believed to be incidental. This is because symptoms at an earlier stage of LC are considered rare and not related to tumor and are largely ignored [4], [5], [6]. Then it is important to understand what makes early stage LC patients seek medical attention.

Because of uncertainty regarding the reasons that caused initial detection, we retrospectively reviewed electronic medical records to collect this information in patients diagnosed with early-stage LC. We are interested in how often LC patients were diagnosed because of symptoms at their early stage and whether these symptoms were related to early-stage lung tumor, and whether the presence of symptoms at early stage compromises survival. Contrary to the accepted theory that early LC is asymptomatic, symptoms was the number one reason that caused initial detection in this study population. Furthermore symptoms showed an association with tumor size at presentation but no association with survival. Although the clinical usefulness of this information is not determined and the results need to be verified by other independent preferably prospective studies, our data indicate that the general belief on asymptomatic early stage LC may have to be reconsidered.

## Methods

### Patients

We retrospectively reviewed the electronic medical records of 1396 patients with stage I or II, out of 4502 all stage NSCLC patients identified through the institutional databases (Tumor Registry and Patients' Hidstory database) who presented between 2000 and 2009 at The University of Texas MD Anderson Cancer Center (Houston, Texas). This study was approved by the M. D. Anderson Cancer Center's Office of Human Research Protections (OHRP) with institutional review board IRB00005015 and a waiver of informed consent because the data are analyzed anonymously. There were no age, gender, or race restrictions. We extracted the following types of data: age, sex, race, smoking status, tumor stage, cell type, and follow-up therapies. The staging was done according to the American Joint Committee on Cancer Staging System. Information on the main reason that made patients seek medical attention and finally led to diagnosis was extracted from the “History of present illness” as recorded by a physician during initial evaluation. Records of tumor size were verified by surgical reports of 961 patients. For our analysis, we used the maximum tumor size dimension.

We collected vital status (dead or alive) by the National Death Index search. The cutoff date for all living patients was July 15, 2010. The survival time was calculated from the date of the LC diagnosis.

### Mode of Detection

**Figure 1:**
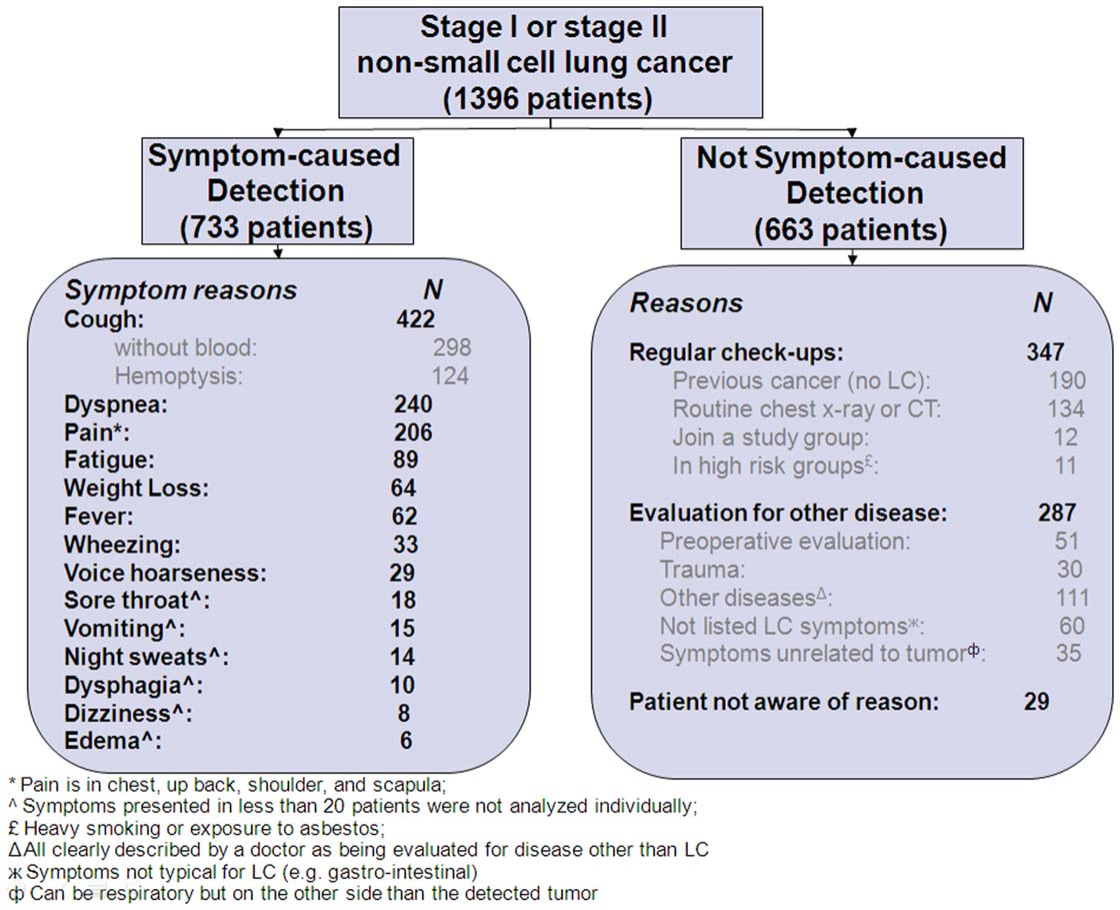
The reasons causing initial medical attention.

We divided the reasons that made patients seek medical attention into two main categories: suggestive LC-symptom-caused (Briefly described as symptom-caused in this article) and not symptom-caused (including regular check-ups, evaluation of other diseases and unknown reasons).(Figure 1) Suggestive LC-symptoms are considered characteristic of LC in literature [7]. Here is an example of how the doctor recorded the information at patient's initial evaluation.

- *The patient, in December, began to have difficulty with breathing, coughing up blood for 4 to 5 weeks, 2 times a week. He was given nebulizers with albuterol which did not resolve. He was then seen by Dr. (omitted), a pulmonologist, who did a chest x-ray which proved to be abnormal. He then underwent a CT scan of the chest on 01/12/05. This revealed a mass in the right upper lobe but no mediastinal adenopathy.*

This IB stage LC patient was assigned to symptom-caused detection group because of the obvious new symptoms which prompted medical attention and led to diagnosis of LC. Reasons other than symptoms are also listed in Figure 1.

We were dealing with “evaluation of other diseases” group (287 patients) as follows. Patients diagnosed with LC while undergoing a preoperative evaluation (for an unrelated condition; n = 50) or an evaluation for a trauma (n = 30) were attributed to the non-symptom-caused detection group. 111 patients in “other disease” group were all clearly described by a doctor as being evaluated for disease other than LC. 60 of the remaining 95 patients presented with symptoms that are not typical for LC (e.g. gastrointestinal). Finally, 35 patients with transient suggestive LC symptoms thought to be unrelated to LC (e.g. those occurring on the opposite side from detected LC), were also classified as having incidentally detected cancer [8]. 29 (2%) patients were not aware of any diseases or symptoms and were seen by a doctor because they: (i) didn't have a check-up for years; (ii) a friend or a family member was diagnosed with cancer; or because (iii) they just wanted a check-up without any obvious reason). 39 patients reported suggestive LC symptoms when they were diagnosed, but these symptoms were not the reason they sought medical care. These patients were also classified into the non-symptom-caused category. We performed sensitivity analyses excluding these patients (about 6%) to reduce the effect of arbitrary grouping.

Patients in symptom-caused detection group had at least one of the suggestive symptoms of LC that are considered characteristic of LC. We subdivided the individual symptoms into two subcategories. The thoracic and throat symptoms category included cough, dyspnea, shoulder, scapula and chest pain, wheezing, dysphagia, sore throat, and voice hoarseness. The general symptoms category included fatigue, night sweat, weight loss, fever, dizziness, vomiting, and edema.

### Statistical Analysis

#### Prevalence of Symptoms

The proportion of symptom-caused detection was evaluated overall and by age group, sex, race and smoking status. We also looked into these groups stratified by stage to find out the relationship between the demographic factors and proportion of symptom-caused detection.

#### The Relationship of Tumor Size and Prevalence of Symptoms

To evaluate the relation between the tumor size and symptoms, we grouped the patients into 10 categories by using deciles of the tumor size distribution, to obtain equal size groups (each group contained about one hundred patients). In each group, the mean tumor size and the proportion of symptom-caused detection were calculated. In stage IA LC specifically, we grouped the patients into 5 categories according to quintiles of the tumor size distribution to illuminate the relationship between symptom-caused detection and tumor size at that stage.

The Student's *t* test, Kruskal-Wallis test, and one-way ANOVA were used to evaluate the associations between tumor size and symptoms. The level of significance was set at *P*<.05. We applied Bonferroni correction when multiple testing was performed. Because of deviation of the tumor size distribution from normality, logarithmic transformation was used to calculate *P* values for surgical maximum size. Univariate analyses using Kaplan-Meier and Cox Regression were performed to determine the effect of age, gender, ethnicity, smoking status, stage status, and the therapy. Multivariate Cox regression was performed to determine the effect of presence of symptoms on overall and LC-specific survival. The multivariate Cox regression model was used to adjust for gender, age, ethnicity, smoking status, stage, and the therapy. All analyses were performed using SPSS software, version 16.0, for Windows.

## Results

### Proportion of Symptom-Caused Detection

In this case series, the most common reason (52.5%) for patients with early-stage NSCLC to seek medical attention and treatment was presence of symptom, followed by regular check-ups (24.9%) and evaluation for other diseases (20.6%). The demographic characteristics of symptom and non-symptom groups are shown in Table 1. In particular, for the stage IA patients, 43.1% went to doctors because of symptoms. The most common complaints were cough (with or without blood), dyspnea (shortness of breath), pain (scapula and chest), fever, sore throat, weight loss, fatigue, wheezing, voice hoarseness, dysphagia, dizziness, edema, vomiting, and night sweats. The thoracic and throat symptoms were most common as chief complaints (91%), followed by general symptoms (fever, fatigue, or weight loss) (24.4%). Individually, cough, dyspnea, and pain (scapula and chest) were the top three reported complaints (cough, 57.6%, dyspnea, 32.7%, and pain, 28.1%).

**Table 1:** Demographic characteristics of patients diagnosed through symptoms and for other reasons.

Characteristics	All (N = 1396)	▵Symptom-caused detection(N = 733)	Other detection(N = 663)	*P* value**
Sex, n (%)				
Male	725 (51.9)	377 (51.4)	348 (52.5)	.693
Female	671 (48.1)	356 (48.6)	315 (47.5)	
*Age, n (%)				
≤68	749 (53.7)	430 (58.7)	319 (48.1)	<.001
>68	647 (46.3)	303 (41.3)	344 (51.9)	
Ethnicity, n (%)				
White	1213 (86.9)	632 (86.2)	581 (87.6)	.286
Black	98 (7.0)	53 (7.2)	45 (6.8)	
Hispanic	58 (4.2)	29 (4.0)	29 (4.4)	
Other	27 (1.9)	19 (2.6)	8 (1.2)	
Stage, n (%)				
IA	613 (43.9)	264 (36.0)	349 (52.6)	<.001
IB	429 (30.7)	245 (33.4)	184 (27.8)	
IIA	88 (6.3)	46 (6.3)	42 (6.3)	
IIB	266 (19.1)	178 (24.3)	88 (13.3)	
Smoking, n (%)				
Never	192 (13.8)	93 (12.7)	99 (15.0)	0.010
Former	713 (51.2)	355 (48.5)	358 (54.2)	
Recent Quitter	167 (12.0)	104 (14.2)	63 (9.5)	
Current	321 (23.0)	180 (24.6)	141 (21.3)	
Cell type, n (%)				
Adenocarcinoma	584 (41.8)	269 (36.7)	315 (47.5)	<.001
Squamous cell carcinoma	406 (29.1)	240 (32.7)	166 (25.0)	
Bronchioloalveolar carcinoma	93 (6.7)	39 (5.3)	54 (8.1)	
Others including mixed types	313 (22.4)	185 (25.2)	128 (19.3)	
Therapy type, n (%)				
No therapy	3 (0.2)	1 (0.1)	2 (0.3)	<.001
Surgery	741 (53.1)	341 (46.5)	400 (60.3)	
Chemo	65 (4.7)	41 (5.6)	24 (3.6)	
Radiation	284 (20.3)	151 (20.6)	133 (20.1)	
Surgery & Chemo	182 (13.0)	115 (15.7)	67 (10.1)	
Surgery & Radiation	25 (1.8)	19 (2.6)	6 (0.9)	
Chemo & Radiation	76 (5.4)	52 (7.1)	24 (3.6)	
Surgery & Chemo & Radiation	20 (1.4)	13 (1.8)	7 (1.1)	

* Age 68 is the median.

** Determined by chi-square test.

▵ Symptoms suggestive of LC.

No difference in the proportion of symptom-caused detection was observed by sex and ethnicity. Younger patients (age ≤68, median) were diagnosed because of symptoms more often compared to older patients (age >68, *P*<.001). Recent quitters and current smokers presented more symptom-caused detection than never and former smokers (*P* = .006). Squamous cell carcinoma caused more symptoms than adenocarcinoma (*P*<.001). Symptomatic patients were less likely to be stage IA and had more combined therapies compared to patients with non-symptom caused detection (*P*<.001). After stratification by stage, there still was no gender or ethnicity related difference in prevalence of symptom-caused detection (Table S1). The difference by age group was observed in stage IA (borderline significant *P* = .049) and IB (*P* = .009). The difference by smoking status and by cell type only existed in stage IA (*P* = .012, and *P* = .016, respectively). The proportion of symptom-caused detection increased along with the stage. We also observed that cough, dyspnea, and pain were the top three reported complaints in all stages.

### Symptoms and Tumor Size

**Figure 2:**
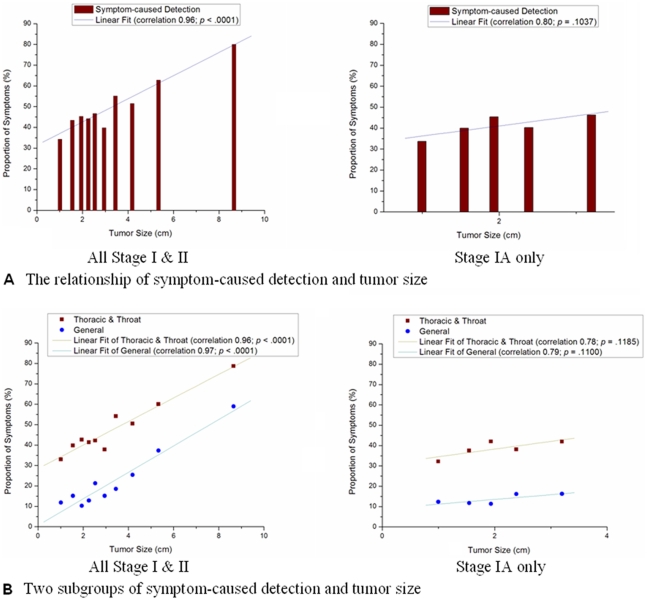
The proportion of symptom-caused detection in each tumor size category: (A) overall symptom, (B) two subgroups of symptoms.

Figure 2A shows that the proportion of symptom-caused detection had a linear relationship with the tumor size (correlation 0.96; with *p*<.001). Figure 2B shows the subgroups of symptom clusters. The proportion of thoracic and throat symptoms and general symptoms both showed a linear relationship with the tumor size. Fever, fatigue, and weight loss were significantly more common in patients with larger tumor size (Figure S1).

The symptom-caused detection group always had significantly larger tumors compared with those of the no symptom-caused detection group (Table S2). However, in stage IA patients the difference of tumor size between symptom-caused detection group and no symptom-caused detection group became insignificant. This is likely due to low variation in the tumor size in stage IA patients (by definition of stage IA). The only exception was cough (without blood) – patients affected by it had significantly larger tumors (*P* = .018) compared with the no symptom-caused detection group in stage IA. The significance of results did not change after sensitivity analysis (after excluding people who had suggestive LC symptoms but resented to a doctor for reasons other than these symptoms). The tumor size (mean: 3.01 cm) of 95 patients who presented with symptoms not suggestive of LC or thought to be unrelated to LC showed no difference (p>.05) from the tumor size in regular check-ups group (mean: 2.69 cm) or from that in the rest of the patients in the group diagnosed through evaluation for other disease (mean: 3.03 cm).

### Symptoms and Survival

The comparison of overall survival time between symptom-caused detection (median, 4.42 years; 95% confidence interval [CI], 3.27–5.56) and non-symptom-caused detection (median: 5.92 years; 95% CI4.44–7.39) showed no significant difference (Log Rank, *P* = .101). After restricting the test to LC-specific survival time, the difference remained not significant (Log Rank, *P* = .483). Figure S2 and Figure S3 show no significant difference in the overall survival and LC specific survival stratified by stage, respectively.

We also compared the survival after adjustment by age, gender, race, smoking status, therapy, and stage, because these factors may affect survival in patients with early-stage NSCLC [9]. Table S3 shows the associations between the overall or LC specific survival and these prognostic factors. All of them were significantly associated with survival and were adjusted for in the final model. Table 2 summarizes the results of the analyses of symptoms as predictors of overall and LC-specific survival after adjustment by age, gender, race, smoking status, therapy, and stage. Only the comparison results of overall symptoms, two subgroups of symptom clusters, and the top three reported individual symptoms (cough, dyspnea, and pain) are shown, as the small case number did not allow for comparisons by other individual symptoms. Our results demonstrated that for the early stages (I and II) of LC, the symptom-caused detection group showed no significant difference in overall and LC-specific survival when compared to the non-symptom-caused detection group. We also tested the proportional hazards assumptions and they were not violated.

**Table 2:** Analysis of symptoms as a predictor of overall and LC specific survival for stage I & II and stage IA only LC after adjustment for age, sex, race, smoking status, stage status, and therapy.

Variable	Overall survival			Lung cancer specific survival		
	HR	95.0% CI	P Value	HR	95.0% CI	P Value
Overall No Symptoms (I & II)	1.000	-	-	1.000	-	-
Overall Symptoms	1.115	0.913–1.361	.284	0.994	0.680–1.454	.977
Thoracic & throat*	1.115	0.910–1.367	.293	1.026	0.698–1.508	.895
Cough(without blood)	1.043	0.919–1.183	.518	1.124	0.898–1.408	.307
Hemoptysis	1.037	0.925–1.162	.534	0.979	0.791–1.212	.846
Dyspnea	1.099	0.958–1.261	.180	1.041	0.809–1.341	.753
Pain	1.162	0.872–1.549	.307	0.915	0.514–1.628	.762
General**	1.251	0.942–1.663	.122	1.180	0.689–2.021	.546
Overall No Symptoms (IA only)	1.000	-	-	1.000	-	-
Overall Symptoms	1.144	0.818–1.600	.443	0.901	0.423–1.920	.788
Thoracic & throat*	1.169	0.829–1.648	.374	1.024	0.477–2.199	.952
Cough(without blood)	1.014	0.806–1.276	.906	1.015	0.626–1.646	.952
Hemoptysis	1.228	0.976–1.546	.080	1.245	0.747–2.076	.401
Dyspnea	1.143	0.908–1.439	.256	1.114	0.720–1.725	.627
Pain	1.173	0.677–2.033	.569	1.198	0.337–4.261	.780
General**	0.995	0.571–1.734	.986	0.368	0.048–2.821	.336

* The thoracic and throat symptoms category included cough, dyspnea, shoulder, scapula and chest pain, wheezing, dysphagia, sore throat, and voice hoarseness.

** The general symptoms category included fatigue, night sweat, weight loss, fever, dizziness, vomiting, and edema.

Variables were coded as 0 for no symptom, 1 for symptom; Age, sex, race, smoking status, stage status, and therapy were adjusted for all stage I & II. Age, sex, race, smoking status, and therapy were adjusted for stage IA by using multivariate Cox Regression.

## Discussion

Contrary to what we expected, detection of early stage (I or II) NSCLC seems not totally incidental. In our study population, 52.5% patients were detected because of symptoms suggestive of a lung tumor. Because of the retrospective design, information on symptoms was extracted from medical records but not directly from interviews or surveys. Thus there might be inaccuracies in the grouping patients into symptom-caused and non-symptom-caused detection, due to different understanding of early LC symptoms. To avoid an arbitrary result, we performed a sensitivity analysis, only including individual most common symptoms: cough, dyspnea and pain, which are accepted LC symptoms. The total number of patients having at least one of these symptoms is 661, still 47.3% of all early stage LC patients.

Our study challenges the general belief that LC is asymptomatic until it reaches an advanced stage. Most suggestive LC symptoms that prompted individuals to seek medical care showed clear association with tumor size at diagnosis, while the non-suggestive LC symptoms or unrelated symptoms didn't. The proportion of symptoms in early stage LC patients is high enough to draw medical attention. Even in stage IA, the earliest stage that is believed to be totally asymptomatic, the proportion of symptoms is still remarkable and the association with tumor size is also observable. Koyi et al. [10] reported that only 7.0% of 364 patients with LC were asymptomatic in their study. Of the 67 (18.4%) patients with stage I or stage II LC in their study, the proportion of symptomatic patients in stage I or stage II was not less than 62.0%, which is even higher than that in our study. Another study by Smith et al. [11] also indicated that LC (including stages III and IV) is almost always symptomatic, usually for several months before consultation.

Although the symptom-caused detection is common in early stage LC patients, these symptoms are usually believed not to be related to LC and largely ignored because they are considered non-specific [4], [12], [13]. However, we observed that the larger tumor size was associated with higher prevalence of symptoms in a close to linear way (Figure 2; *P*<0.0001, Symptoms that are not suggestive of LC or unrelated to LC did not show such an association), which implies that these symptoms might be indicators of the early stage LC. Squamous cell lung carcinoma usually starts near a central bronchus [14], which is likely to cause more symptoms than peripheral LC (such as adenocarcinoma). This is what was observed in our study: SqCC caused more symptoms than. We also found the highest proportion of symptom-caused detection among recent quitters, consistent with the previous observation that ex-smokers within one year of cessation have high standardized lung cancer mortality ratios. This phenomenon may be explained by the so-called reverse causality bias – symptoms compel smokers to quit, although smokers attribute these symptoms to smoking rather than developing lung cancer [15], [16] Recent quitters and current smokers have a higher proportion of symptom-caused detection than never and former smokers. This implies that long standing, habitual smoking-related symptoms do not prevent smoking patients from noticing new symptoms, indicating existence of specific early changes in their typical symptoms.

**Figure 3:**
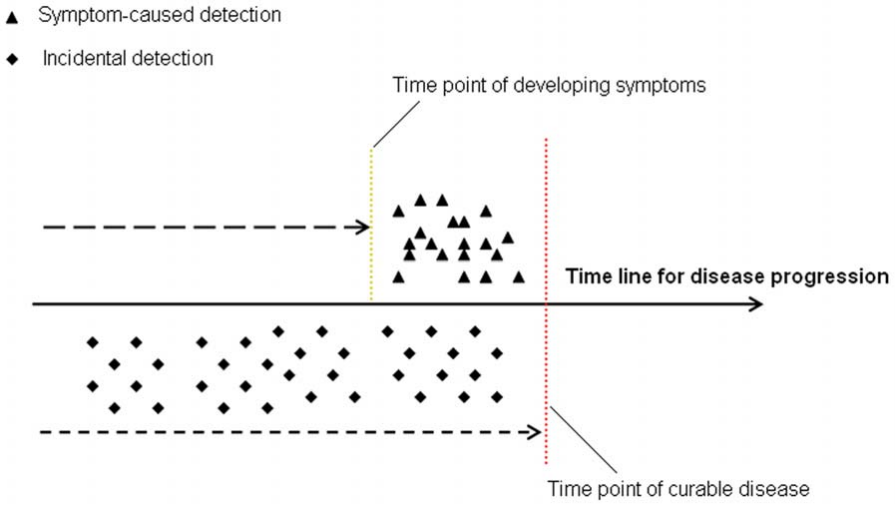
Interpretation of tumor size difference and survival difference in symptom-caused detection and non-symptom-caused detection.

Symptom and non-symptom diagnosed patients showed similar survival even if stratified by stage. One can suggest that there is a time point when LC from curable becomes incurable, and also a time point of symptom development. If the turning point of curable to incurable LC occurs after symptom development, there exists an opportunity time window, during which an appropriate intervention can result in a cure. (Figure 3) This is consistent with both the observation of larger tumor sizes at diagnosis and worse stage distribution in symptom-caused versus non-symptom-caused detected patients, and with the fact that survival does not show much difference between these two groups.

A study by Corner et al. [5] indicated that people would not pay attention to symptoms until the problem was so severe that it could no longer be tolerated. In another study, Smith et al. performed a cross-sectional quantitative interview survey of 360 patients with newly diagnosed primary LC in three Scottish hospitals [11], and concluded that the time between symptom onset and consultation was long enough to plausibly affect prognosis [11]. In our study the average time is about 2 months, which may partially explain the larger tumor size in the symptom-caused detection group. Smith et al. also found that hemoptysis, new onset of shortness of breath, cough, and loss of appetite were significantly associated with earlier consulting. Those findings are also consistent with our findings that the main symptoms in early stage NSCLC were cough and dyspnea.

Naruke et al [17] compared the survival of screen-detected versus symptom-detected LC patients for all stages (I–IV) in Japan. The symptom-detected group had significantly lower survival than did the screen-detected group [17]. Because advanced patients naturally have more symptoms, the results on survival were confounded by stage and do not explain the situation of the LC at a curable stage (stage I & II). A different study from Japan by Sobue et al. [18] focused on stage I LC patients without surgical treatment and also observed a poorer survival in symptomatic versus asymptomatic patients. However, without the surgical treatment, which is the most efficient treatment for early-stage NSCLC, survival will depend on the extent of tumor progression. Thus, the result is in agreement with our finding that the presence of symptoms is related to a larger, likely more progressed tumor at the time of diagnosis. (Figure 3) Another study by Raz et al. [8] found no overall difference in stage-adjusted survival between symptomatic LC and incidental detection, although they might not have had statistical power to detect the difference due to the limited sample size with 100 incidentally detected and 174 symptom detected patients.

Our study might be the largest retrospective study on symptoms in early stage LC. The major implication is that symptoms may be a potential alerting indicator for early-stage NSCLC but will not necessarily compromise survival. A study in ovarian cancer, previously believed to be asymptomatic, demonstrated that symptoms can be used to diagnose ovarian cancer earlier (Goff et al.). [19], [20] Our data can thus be used in a model for prediction of early-stage NSCLC.

Unlike Goff et al.'s study, our study was limited in that our subjects were only LC patients, but controls (high-risk individuals without LC) were not available. Acquiring the group of controls should be the next step to determine whether the symptoms are useful for screening in a population of high risk for LC (e.g. older smokers). Another limitation of our study is that symptoms identified retrospectively may be incomplete and arbitrary. Although we performed data extraction carefully, we cannot rule out the variations in documenting symptoms by different doctors at the time of initial evaluation. Thus, an independent prospective study is needed to verify our results.

Our findings suggest that the general belief that early LC is asymptomatic should be rethought carefully, and guidelines for earlier recognition of LC are well worth of further study. Due to NLST results, there is a renewed interest in lung cancer screening and an urgent need to identify highest risk group that should be screened. Potentially the symptom based alert system, in combination with known lung cancer risk factors, can help identify such a group, which will increase the yield of cases and improve screening efficiency.

## Supporting Information

Figure S1 **The proportion of symptom-caused detection in each tumor size category for individual symptoms.**
(TIF)

Figure S2 **Overall survival for patients (stratified by stage).**
(TIF)

Figure S3 **LC-specific survival for patients (stratified by stage).**
(TIF)

Table S1 Demographic characteristics of patients diagnosed through symptoms and for other reasons, by stage.
(DOCX)

Table S2 Tumor size in patients diagnosed through symptoms and for other reasons.
(DOCX)

Table S3 Univariate analysis of factors as predictors of overall and LC specific survival.
(DOCX)
