# Supplementary figures and images for: Initial Medical Attention on Patients with Early-Stage Non-Small Cell Lung Cancer
Source: PLoS One. 2012 Mar 7;7(3):e32644. doi: 10.1371/journal.pone.0032644 (PMC3296738; doi:10.1371/journal.pone.0032644)

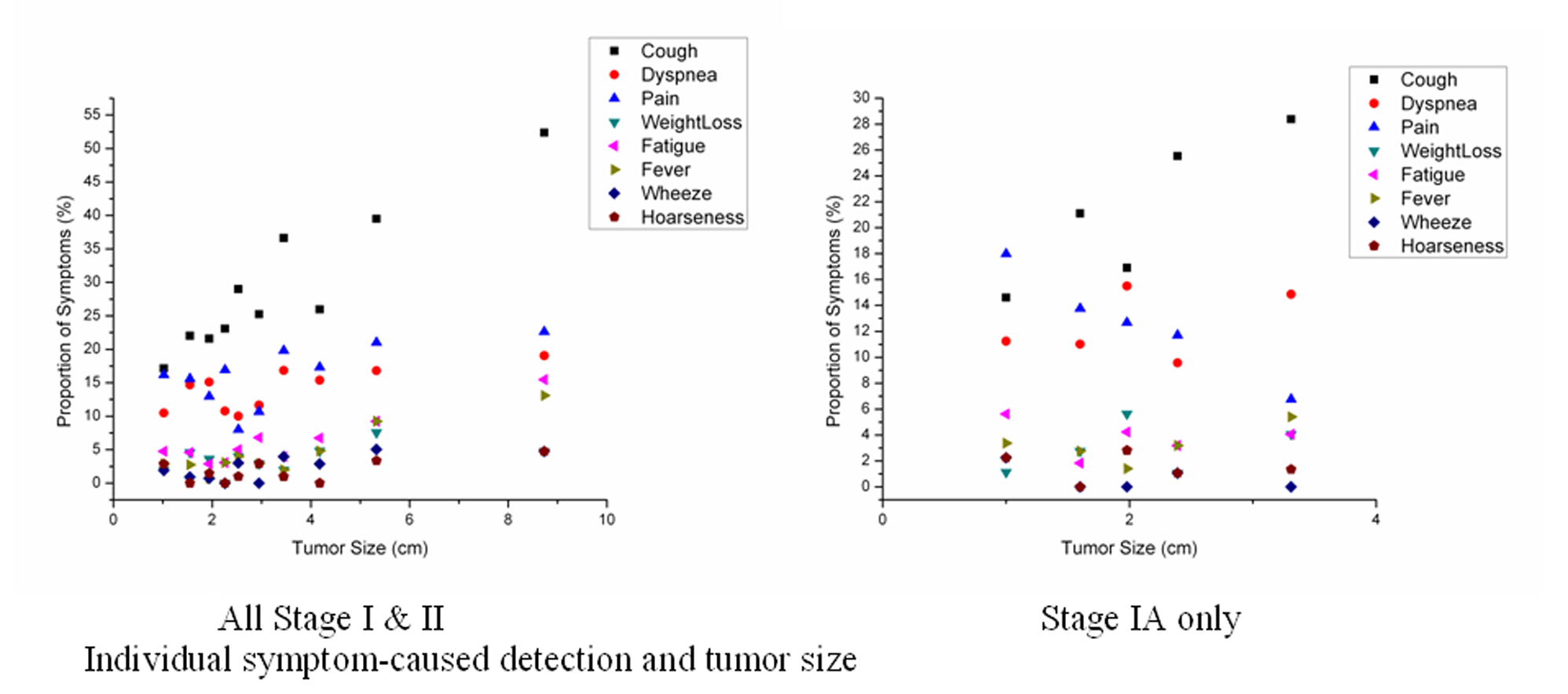

Supplement: Figure S1 — The proportion of symptom-caused detection in each tumor size category for individual symptoms. (TIF) [file pone.0032644.s001.tif]

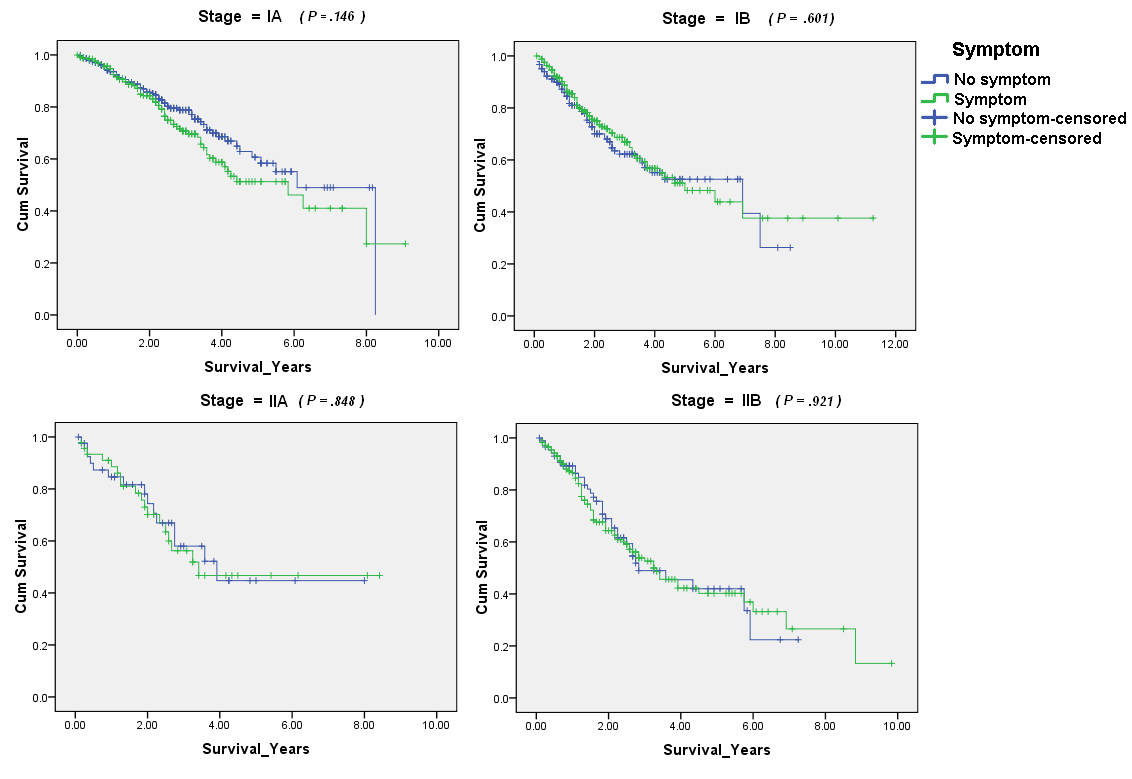

Supplement: Figure S2 — Overall survival for patients (stratified by stage). (TIF) [file pone.0032644.s002.tif]

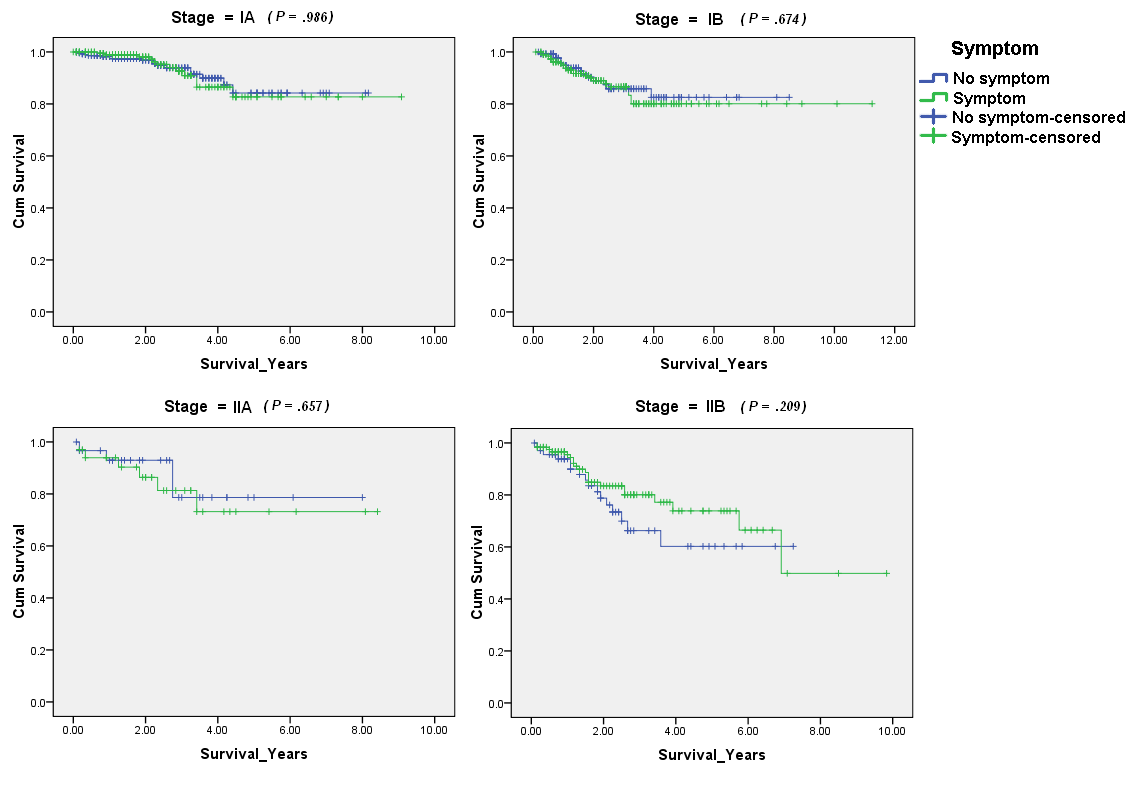

Supplement: Figure S3 — LC-specific survival for patients (stratified by stage). (TIF) [file pone.0032644.s003.tif]
