# Supplementary material for: Initial Medical Attention on Patients with Early-Stage Non-Small Cell Lung Cancer
Source: PLoS One. 2012 Mar 7;7(3):e32644. doi: 10.1371/journal.pone.0032644 (PMC3296738; doi:10.1371/journal.pone.0032644)
Supplement: Table S1 — Demographic characteristics of patients diagnosed through symptoms and for other reasons, by stage. (DOCX) [file pone.0032644.s004.docx]

**Table S1** Demographic characteristics of patients diagnosed through symptoms and for other reasons, by stage

| **Characteristic** | **Symptom-caused detection of all lung cancer patients *n/N* (%) by Stage** | | | | | | | | |
| --- | --- | --- | --- | --- | --- | --- | --- | --- | --- |
|  | **Total I & II** | **IA** | ***P* value** | **IB** | ***P* value** | **IIA** | ***P* value** | **IIB** | ***P* value** |
| Total | 733/1396 (52.5) | 264/613 (43.1) |  | 245/429 (57.1) |  | 46/88 (52.3) |  | 178/266 (66.9) |  |
| Sex |  |  |  |  |  |  |  |  |  |
| Male | 377/725 (52.0) | 123/293 (42.0) | .603 | 121/222 (54.5) | .259 | 24/47 (51.1) | .808 | 109/163 (66.9) | .984 |
| Female | 356/671 (53.1) | 141/320 (44.1) |  | 124/207 (59.9) |  | 22/41 (53.7) |  | 69/103 (67.0) |  |
| Age |  |  |  |  |  |  |  |  |  |
| ≤68 | 430/749 (57.4) | 143/304 (47.0) | .049 | 139/220 (63.2) | .009 | 32/60 (53.3) | .771 | 116/165 (70.3) | .134 |
| >68 | 303/647 (46.8) | 121/309 (39.2) |  | 106/209 (50.7) |  | 14/28 (50.0) |  | 62/101 (61.4) |  |
| Ethnicity |  |  |  |  |  |  |  |  |  |
| White | 632/1213 (52.1) | 232/540 (43.0) | .323 | 209/378 (55.3) | .146 | 41/75 (54.7) | .395 | 150/220 (68.2) | .579 |
| African-American | 53/98 (54.1) | 16/38 (42.1) |  | 17/22 (77.3) |  | 3/8 (37.5) |  | 17/30 (56.7) |  |
| Hispanic | 29/58 (50.0) | 9/25 (36.0) |  | 9/15 (60.0) |  | 1/4 (25.0) |  | 10/14 (71.4) |  |
| Smoking |  |  |  |  |  |  |  |  |  |
| Never | 93/192 (48.4) | 44/109 (40.4) | .012 | 34/58 (58.6) | .846 | 3/8 (37.5) | .362 | 12/17 (70.6) | .764 |
| Former | 355/713 (49.8) | 117/300 (39.0) |  | 132/235 (56.2) |  | 23/49 (46.9) |  | 83/129 (64.3) |  |
| Recent Quitter | 104/167 (62.3) | 40/66 (60.6) |  | 22/41 (53.7) |  | 8/13 (61.5) |  | 34/47 (72.3) |  |
| Current | 180/321 (56.1) | 62/137 (45.3) |  | 57/94 (60.6) |  | 12/18 (66.7) |  | 49/72 (68.1) |  |
| Cell type |  |  |  |  |  |  |  |  |  |
| Adenocarcinoma | 269/584 (46.1) | 110/283 (38.9) | .016 | 91/174 (52.3) | .238 | 13/34 (38.2) | .074 | 55/93 (59.1) | .231 |
| Squamous cell carcinoma | 240/406 (59.1) | 68/150 (45.3) |  | 82/130 (63.1) |  | 20/30 (66.7) |  | 70/96 (72.9) |  |
| Bronchioloalveolar carcinoma | 39/93 (41.9) | 19/56 (33.9) |  | 17/33 (51.5) |  | - |  | 3/4 (75.0) |  |
| Others including mixed types | 185/313 (59.1) | 67/124 (54.0) |  | 55/92 (59.8) |  | 13/24 (54.2) |  | 50/73 (68.5) |  |
| Therapy type |  |  |  |  |  |  |  |  |  |
| No therapy | 1/3 (33.3) | 0/1 (0.0) | .176 | 0/1 (0.0) | .170 | 1/1 (100.0) | .792 | - | .147 |
| Surgery | 341/741 (46.0) | 145/369 (39.3) |  | 117/227 (51.5) |  | 13/31 (41.9) |  | 66/114 (57.9) |  |
| Chemo | 41/65 (63.1) | 8/13 (61.5) |  | 10/17 (58.8) |  | 7/13 (53.8) |  | 16/22 (72.7) |  |
| Radiation | 151/284 (53.2) | 88/183 (48.1) |  | 45/74 (60.8) |  | 3/5 (60.0) |  | 15/22 (68.2) |  |
| Surgery & Chemo | 115/182 (63.2) | 6/15 (40.0) |  | 49/77 (63.6) |  | 13/24 (54.2) |  | 47/66 (71.2) |  |
| Surgery & Radiation | 19/25 (76.0) | 7/11 (63.6) |  | 7/8 (87.5) |  | 1/1 (100.0) |  | 4/5 (80.0) |  |
| Chemo & Radiation | 52/76 (68.4) | 7/17 (41.2) |  | 12/17 (70.6) |  | 7/11 (63.6) |  | 26/31 (83.9) |  |
| Surgery & Chemo & Radiation | 13/20 (65.0) | 3/4 (75.0) |  | 5/8 (62.5) |  | 1/2 (50.0) |  | 4/6 (66.7) |  |
