# Supplementary material for: Initial Medical Attention on Patients with Early-Stage Non-Small Cell Lung Cancer
Source: PLoS One. 2012 Mar 7;7(3):e32644. doi: 10.1371/journal.pone.0032644 (PMC3296738; doi:10.1371/journal.pone.0032644)
Supplement: Table S2 — Tumor size in patients diagnosed through symptoms and for other reasons. (DOCX) [file pone.0032644.s005.docx]

**Table S2** Tumor size in patients diagnosed through symptoms and for other reasons

| **Symptoms** | | **All Stage I & II** | | | | **Only Stage IA** | | | |
| --- | --- | --- | --- | --- | --- | --- | --- | --- | --- |
|  |  | **N** | **Median/mean, cm** | **IQR/SD** | ***P* median/*P* mean** | **N** | **Median/mean, cm** | **IQR/SD** | ***P* median/*P* mean** |
| Overall No Symptoms* | | 480 | 2.50/2.82 | 1.70-3.50/1.66 |  | 237 | 1.90/1.92 | 1.40-2.50/0.76 |  |
| Overall Symptoms | | 481 | 3.00/3.80 | 2.00-5.00/2.56 | <.001/<.001 | 164 | 2.00/2.05 | 1.50-2.50/0.92 | .206/.139 |
| Thoracic & Throat | | 437 | 3.10/3.84 | 2.00-5.00/2.61 | <.001/<.001 | 146 | 2.00/2.04 | 1.50-2.50/0.95 | .298/.174 |
|  | Cough |  |  |  |  |  |  |  |  |
|  | Yes, without blood | 193 | 3.10/3.94 | 2.00-5.00/2.87 | <.001/<.001 | 64 | 2.15/2.26 | 1.50-2.65/1.20 | .031/.033 |
|  | Hemoptysis | 84 | 3.90/4.27 | 2.50-5.50/2.18 | <.001/<.001 | 18 | 1.75/1.89 | 1.40-2.50/0.70 | .935/.875 |
|  | Dyspnea |  |  |  |  |  |  |  |  |
|  |  | 137 | 3.00/3.70 | 2.00-4.65/2.48 | <.001/<.001 | 47 | 2.00/2.13 | 1.40-2.50/1.34 | .616/.138 |
|  | Pain |  |  |  |  |  |  |  |  |
|  | Shoulder, scapular | 31 | 4.50/5.17 | 2.00-6.50/3.96 | <.001/<.001 | 5 | 1.95/1.72 | 1.20-2.15/0.52 | .562/.449 |
|  | Chest, upper back | 122 | 3.00/3.41 | 1.80-4.23/2.38 | .019/.002 | 48 | 1.75/1.78 | 1.20-2.20/0.70 | .205/.220 |
|  |  |  |  |  |  |  |  |  |  |
|  | Wheezing | 22 | 4.00/4.27 | 2.50-5.00/2.43 | .001/<.001 | 3 | 1.10/1.40 | 0.60-./0.98 | .281/.460 |
|  |  |  |  |  |  |  |  |  |  |
|  | Voice hoarseness | 18 | 3.25/4.14 | 2.00-5.75/2.58 | .027/.001 | 6 | 2.00/1.87 | 0.93-2.63/0.88 | .986/.895 |
|  |  |  |  |  |  |  |  |  |  |
| General | | 129 | 3.70/4.22 | 2.45-5.50/2.46 | <.001/<.001 | 37 | 2.00/2.08 | 1.40-2.50/0.96 | .484/.345 |
|  |  |  |  |  |  |  |  |  |  |
|  | Fever | 41 | 4.90/4.89 | 2.70-6.50/2.60 | <.001/<.001 | 11 | 2.30/2.25 | 1.20-2.70/1.44 | .639/.182 |
|  | Weight Loss | 35 | 3.50/3.84 | 2.00-5.00/2.23 | .005/.001 | 10 | 2.00/2.25 | 1.75-2.70/1.00 | .250/.317 |
|  | Fatigue | 57 | 4.00/4.47 | 2.50-5.75/2.70 | <.001/<.001 | 11 | 1.50/1.67 | 1.00-2.50/0.72 | .317/.296 |
|  |  |  |  |  |  |  |  |  |  |

*All comparison are with the no symptoms group (non-symptom-caused detection), 39 symptomatic people who ignored symptoms are among incidental group. While excluding the 39 symptomatic people from the incidental group, the median tumor size and IQR of incidental group (all stage I & II) did not change and the mean / SD changed to 2.82 cm / 1.69. For stage IA only, the median size and IQR did not change either, the mean / SD changed to 1.97 cm / 1.04. The statistic significance did not change in all comparisons.

Tumor size was verified by surgical report in 1029 patients.
