# Supplementary material for: Initial Medical Attention on Patients with Early-Stage Non-Small Cell Lung Cancer
Source: PLoS One. 2012 Mar 7;7(3):e32644. doi: 10.1371/journal.pone.0032644 (PMC3296738; doi:10.1371/journal.pone.0032644)
Supplement: Table S3 — Univariate analysis of factors as predictors of overall and LC specific survival. (DOCX) [file pone.0032644.s006.docx]

**Table S3** Univariate analysis of factors as predictors of overall and LC specific survival

| **Categorical variables*** | **Overall survival** | | | **Lung cancer specific survival** | | |
| --- | --- | --- | --- | --- | --- | --- |
|  | Mean (year) | 95.0% CI | Log Rank | Mean (year) | 95.0% CI | Log Rank |
| Gender |  |  | < .001 |  |  |  |
| Male | 5.471 | 4.822-6.120 |  | 8.920 | 8.089-9.750 | .020 |
| Female | 5.642 | 5.225-6.059 |  | 7.825 | 7.454-8.196 |  |
| Ethnicity |  |  | .527 |  |  | .030 |
| White | 6.041 | 5.524-6.559 |  | 9.375 | 8.911-9.839 |  |
| Black | 4.476 | 3.700-5.252 |  | 5.463 | 4.759-6.168 |  |
| Hispanic | 4.504 | 3.557-5.450 |  | 5.792 | 4.866-6.717 |  |
| Other | 4.462 | 3.277-5.647 |  | 3.679 | 3.385-3.974 |  |
| Stage |  |  | < .001 |  |  | < .001 |
| IA | 5.655 | 5.171-6.139 |  | 8.095 | 7.714-8.476 |  |
| IB | 6.035 | 5.259-6.811 |  | 9.497 | 8.946-10.047 |  |
| IIA | 4.935 | 4.069-5.800 |  | 6.866 | 5.996-7.736 |  |
| IIB | 4.479 | 3.851-5.107 |  | 6.813 | 5.833-7.793 |  |
| Smoking |  |  | < .001 |  |  | .030 |
| Never | 6.428 | 5.623-7.232 |  | 7.705 | 6.934-8.477 |  |
| Former | 5.978 | 5.372-6.584 |  | 9.347 | 8.721-9.972 |  |
| Recent Quitter | 5.824 | 5.069-6.579 |  | 7.356 | 6.886-7.825 |  |
| Current | 4.808 | 4.171-5.446 |  | 7.622 | 6.909-8.335 |  |
| Therapy type |  |  | < .001 |  |  | < .001 |
| No therapy | 2.111 | 0.389-3.833 |  | - | - |  |
| Surgery | 6.364 | 5.830-6.898 |  | 9.035 | 8.736-9.334 |  |
| Chemo | 3.255 | 2.492-4.018 |  | 5.056 | 3.950-6.162 |  |
| Radiation | 3.091 | 2.602-3.580 |  | 3.686 | 3.438-3.934 |  |
| Surgery & Chemo | 6.687 | 5.505-7.869 |  | 8.622 | 7.206-10.039 |  |
| Surgery & Radiation | 3.509 | 2.447-4.570 |  | 4.530 | 3.315-5.746 |  |
| Chemo & Radiation | 2.998 | 2.456-3.540 |  | 4.531 | 3.766-5.296 |  |
| Surgery & Chemo & Radiation | 4.889 | 2.909-6.868 |  | 6.028 | 3.872-8.185 |  |
|  |  |  |  |  |  |  |
| **Continuous variable**** | HR |  | *P* value | HR |  | *P* value |
| Age | 1.037 | 1.026-1.048 | < .001 | 1.031 | 1.011-1.051 | .002 |

* Categorical variables were analyzed by using Kaplan-Meier. ** Continuous variables were analyzed by using Cox Regression.
